# Corneal sensory nerve loss induced by repeated subconjunctival and topical bupivacaine disrupts tear secretion and enhances bacterial adhesion via neuropeptide modulation

**DOI:** 10.1371/journal.pone.0329112

**Published:** 2025-08-04

**Authors:** Ananya Datta, Grace Kelly Orallo, Nahomy Nelson

**Affiliations:** New England College of Optometry Boston, Boston, Massachusetts, United States of America; Harvard Medical School, UNITED STATES OF AMERICA

## Abstract

**Purpose:**

Corneal sensory innervation plays a crucial role in maintaining ocular surface integrity and immune homeostasis by regulating neuropeptide secretion in tear fluid. Sensory dysfunction disrupts tear production and neuropeptide signaling, increasing susceptibility to microbial infections. However, the mechanistic link between sensory nerve suppression, neuropeptide depletion, and bacterial adhesion remains incompletely understood. This study establishes a refined protocol for targeted corneal sensory nerve suppression using bupivacaine, a long-acting local anesthetic, and investigates the roles of substance P (SP) and calcitonin gene-related peptide (CGRP) in modulating tear production and bacterial adhesion.

**Method:**

Male and female C57BL/6J (wild-type) mice (6–8 weeks old) were used to establish a localized and sustained corneal nerve suppression model via subconjunctival bupivacaine injection combined with topical application every other day for 15 days. This approach ensured precise modulation of corneal sensory function. Using this model, we investigated how sensory denervation influences microbial adhesion dynamics for *Pseudomonas aeruginosa, Staphylococcus aureus,* and *Staphylococcus epidermidis*, three clinically relevant pathogens with distinct adhesion mechanisms. Bacterial inoculation was standardized using the Kimwipe blotting method to achieve uniform deposition onto the corneal surface, followed by quantification of bacterial adhesion. Tear production was assessed using SMTube testing to evaluate nerve depletion-associated alterations. Enzyme-linked immunosorbent assay (ELISA) was used to quantify SP and CGRP levels in tear fluid, determining whether their depletion correlated with increased bacterial adhesion and altered tear production. To assess whether neuropeptide restoration mitigates bacterial adhesion, SP, CGRP, or phosphate-buffered saline (PBS; control) was administered via subconjunctival injection prior to bupivacaine treatment on day 14 and 15 during the experimental timeline. All assessments, including nerve depletion effects on tear production, bacterial adhesion, and neuropeptide loss, were conducted on day 15 post-bupivacaine treatment.

**Results:**

Targeted corneal sensory denervation via combined subconjunctival and topical bupivacaine resulted in a ~ 50% reduction in corneal nerve density, achieving deeper and more localized nerve suppression compared to subconjunctival injection alone (P < 0.0001). This approach led to a 2.3-fold (~56.6%) reduction in tear production without inducing epithelial damage (P < 0.0001). This loss of sensory input led to a marked decrease in SP and CGRP levels in both the cornea and tear fluid, with the most pronounced reduction observed in the combined treatment group. Notably, neuropeptide depletion correlated with increased bacterial adhesion, with a ~ 1.18-fold increase for *S. aureus* and ~1.20-fold for *P. aeruginosa*, highlighting the critical role of corneal sensory nerves in modulating ocular surface immunity (P < 0.0001). Exogenous SP or CGRP supplementation restored neuropeptide levels and CGRP supplementation reversed bacterial adhesion, highlighting their critical function in maintaining antimicrobial defense.

**Conclusion:**

This study establishes a novel, controlled model of corneal sensory denervation, revealing a direct link between neuropeptide depletion, impaired tear production, and increased microbial adhesion. By simulating neuropathic conditions such as diabetic keratopathy and neurotrophic keratitis, this approach provides a valuable framework for investigating neuroimmune interactions in ocular infections. Beyond infection models, this subconjunctival injection strategy serves as a versatile platform for studying ocular drug pharmacokinetics, neuroprotective interventions, and immune modulation.

## Introduction

The corneal sensory innervation, primarily derived from the ophthalmic branch of the trigeminal nerve, plays a critical role in maintaining ocular surface homeostasis by integrating structural integrity and immune defense through the dynamic release of neuropeptides [1]. Trigeminal sensory nerve fibers continuously secrete neuropeptides such as substance P (SP) and calcitonin gene-related peptide (CGRP), which are synthesized in the trigeminal ganglion to supply either the corneal surface or lacrimal gland. These neuropeptides exert dual functions—promoting epithelial proliferation and wound healing while modulating immune responses to sustain an optimal balance between defense and tolerance [2,3]. SP enhances goblet cell function and mucin secretion, stabilizing the tear film [4], whereas CGRP limits excessive neutrophil infiltration, preventing chronic inflammation [5]. This neuroimmune interplay underscores the cornea’s reliance on intact trigeminal innervation to resist environmental stressors and microbial challenges.

Disruption of corneal sensory function triggers a cascade of pathological changes that compromise ocular surface immunity and increase susceptibility to microbial infections. In conditions such as diabetic neuropathy and neurotrophic keratitis—both characterized by sensory nerve dysfunction and neuropeptide depletion—this impairment is closely linked to recurrent bacterial keratitis [3,6–9]. Experimental models further demonstrate that sensory denervation downregulates antimicrobial peptide expression (e.g., β-defensins) and disrupts epithelial barrier integrity, facilitating pathogen invasion [10,11]. Notably, sensory nerve loss has been associated with increased adhesion of clinically relevant pathogens, including *Pseudomonas aeruginosa*, *Staphylococcus aureus*, and *Staphylococcus epidermidis*, suggesting a direct or indirect role of corneal sensory nerves in microbial colonization [12,13].

These pathogens employ distinct adhesion strategies critical to corneal infections. *P. aeruginosa*, a highly virulent Gram-negative bacterium, is a major cause of microbial keratitis, particularly in contact lens wearers and individuals with corneal surface defects. Its ability to adhere to and invade the epithelium is mediated by pili, flagella, and secreted virulence factors, promoting rapid colonization [14,15]. In contrast, *S. epidermidis*, a Gram-positive commensal bacterium, exploits surface irregularities and altered tear composition to establish biofilms, leading to persistent infections [16,17]. The selection of these bacterial species allows for a comparative assessment of how corneal sensory nerve suppression influences distinct adhesion mechanisms under different pathological conditions. Despite these insights, the molecular mechanisms linking nerve dysfunction to bacterial colonization remain poorly understood.

A critical knowledge gap exists regarding how sensory nerve suppression and neuropeptide depletion modulate bacterial adhesion on the ocular surface. While previous studies have shown that anesthetic-induced nerve blockade reduces SP and CGRP levels in tears, the downstream effects on tear production and epithelial microbial receptors—such as glycocalyx components or integrins—remain unexplored [18,19]. It remains unclear whether neuropeptides directly inhibit bacterial adhesion through receptor antagonism or indirectly modulate tear production, although the anti-inflammatory and antimicrobial properties of these neuropeptides have been investigated in vitro [20,21]. This gap in understanding limits the development of targeted interventions to restore ocular surface immunity in neuropathic patients.

To address this, our study introduces a refined model of targeted corneal sensory nerve suppression using bupivacaine, a long-acting sodium channel blocker that mimics clinical neuropathy without causing permanent nerve damage. By integrating this pharmacological model with bacterial adhesion assays, we recapitulate key pathophysiological features of diabetic keratopathy and neurotrophic keratitis, providing a controlled platform to dissect neuroimmune interactions in real time. This approach enables precise manipulation of neuropeptide levels and bacterial exposure, allowing us to delineate their individual and synergistic roles in infection susceptibility.

Specifically, we investigate whether SP and CGRP depletion due to sensory nerve suppression alters tear production and epithelial surface properties, thereby enhancing bacterial adhesion. Using quantitative proteomics, we profile neuropeptide levels in tear fluid post-bupivacaine treatment, while scanning electron microscopy and bacterial viability assays assess bacterial adhesion and biofilm formation. Additionally, exogenous SP/CGRP supplementation experiments aim to rescue the neurogenic immune phenotype, offering mechanistic insights into potential therapeutic interventions. By elucidating the interplay between corneal sensory nerves and microbial defense, this study lays the foundation for novel strategies to mitigate infection risks in neuropathic corneas.

## Method

All animal procedures complied with the guidelines set forth by the Association for Research in Vision and Ophthalmology (ARVO) and were approved by the Institutional Animal Care and Use Committee (IACUC) at the New England College of Optometry. The study also adhered to the Public Health Service (PHS) policy on the humane care and use of laboratory animals. Male and female C57BL/6J (wild-type) mice, aged six to eight weeks, were obtained from The Jackson Laboratory and maintained under a 12-hour light/dark cycle with unrestricted access to food and water. Anesthesia was induced using 2–3% isoflurane, ensuring that all procedures were completed within 15 minutes. Although no procedures were designed to cause distress, any mice displaying signs of suffering were promptly euthanized in a humane manner. Euthanasia was conducted by administering deep anesthesia with 4% isoflurane, followed by cervical dislocation prior to enucleation.

### Animal preparation for subconjunctival injection

Mice were anesthetized with 2–3% isoflurane in oxygen using a calibrated vaporizer, with anesthesia depth assessed via the absence of reflexive responses to gentle toe pinching. To minimize systemic anesthetic exposure, all procedures, including subconjunctival injections and topical applications, were completed within 15 minutes. Although the protocol did not involve distress-inducing procedures, animals exhibiting signs of severe discomfort or distress were promptly euthanized. All procedures adhered to institutional animal care guidelines and were approved by the IACUC.

### Corneal nerve block via subconjunctival bupivacaine administration

To achieve targeted suppression of corneal sensory nerves, a combined subconjunctival injection and topical application of bupivacaine were utilized. A 0.5% bupivacaine hydrochloride solution (Pfizer INC. USA) was prepared under sterile conditions, and 5 μL was delivered into the subconjunctival space using a Hamilton 10-μL syringe fitted with a 33-gauge small hub blunt needle (point style 3) [13]. The injection was administered at a 15–20° angle to ensure optimal distribution while minimizing reflux. Immediately following the injection, an additional 5 μL of bupivacaine was topically applied to the central cornea to reinforce surface nerve desensitization. Mice in the control group received equivalent volumes of phosphate-buffered saline (PBS) via both subconjunctival and topical routes to control for injection-related effects.

**Fig 1:**
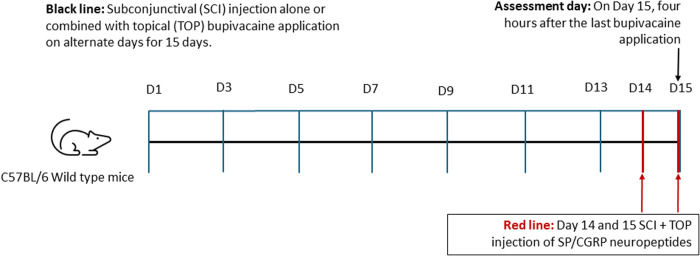
Schematic representation of the timeline for corneal sensory nerve suppression and neuropeptide treatment. Mice received subconjunctival injections (SCI) of 0.5% bupivacaine (BUP; 5 μL) using a Hamilton syringe, followed by topical (TOP) application (5 μL) to the central cornea every other day for 15 days to achieve sustained sensory nerve suppression (black line). Control mice received equivalent volumes of phosphate-buffered saline (PBS) via both routes. In a separate experimental group, neuropeptides CGRP (10.5 μM) or SP (15 μM) were administered subconjunctivally 24 hours before the final bupivacaine treatment (day 14) and again one hour prior to the final bupivacaine injection on day 15 (red line). All assessments, including tear production, fluorescein staining, corneal nerve density, corneal and tear fluid neuropeptide levels, and bacterial adhesion, were conducted four hours after the last bupivacaine injection on day 15.

To sustain sensory nerve suppression while minimizing toxicity, bupivacaine was administered every other day for 15 days. Then on day 15 mice were euthanized using 5% isoflurane in oxygen, followed by cervical dislocation. Corneas were harvested for nerve density analysis or bacterial adhesion assessment and other assessments as mentioned in the Fig 1.

### Neuropeptide treatment in bupivacaine model

In an additional experimental group, calcitonin gene-related peptide (CGRP; 10.5 μM, Cat. No. C0292; Sigma-Aldrich, St. Louis, MO, USA) or substance P (SP; 15 μM, Cat. No. 05-23-0600; Sigma-Aldrich, St. Louis, MO, USA) was administered via subconjunctival injection. The first injection was given 24 hours before bupivacaine treatment on day 14, followed by a second injection one hour before evaluating tear production and bacterial adhesion on day 15 (Fig 1).

### Assessment of aqueous tear production using tear meniscometry

Tear production was evaluated on day 15 post-bupivacaine treatment after 4 hours using the SMTube Testing (SMTM), a refined version of the original SMTube product. The protocol was adapted from previous studies [22,23] and modified to align with the study design. A single SMTM strip, designed to enhance tear absorption through capillary action, was carefully positioned at the inferior tear meniscus of each eye, ensuring gentle contact with the ocular surface and eyelid. The measurement was standardized to a 10-second duration, which was optimized based on human testing parameters while accounting for the lower tear volume in mice. An electronic metronome was used to maintain precise timing. To ensure consistency and minimize variability, all SMTM measurements were performed by the same examiner.

### Corneal fluorescein staining

Corneal epithelial integrity was assessed via fluorescein staining on day 15 after 4 hours post-bupivacaine injection. A 0.5-μL aliquot of 5% sodium fluorescein (Cat. No. 67884; Sigma-Aldrich, St. Louis, MO, USA) was instilled into the inferior conjunctival sac of the right eye using a micropipette, following established protocols [13,24]. After three minutes, corneal staining patterns were examined using a slit lamp biomicroscope under cobalt blue illumination. Punctate staining was evaluated in a masked manner using the National Eye Institute (NEI) grading scale, which scores five predefined corneal regions from 0 to 3, yielding a total score ranging from 0 to 15 [25].

### Animal preparation for bacterial inoculation

Mice were anesthetized via intraperitoneal injection of ketamine (80–100 mg/kg) and dexmedetomidine (0.25–0.5 mg/kg), with anesthesia depth confirmed by the absence of reflexive responses to toe pinch. On day 15, four hours after the final bupivacaine treatment, the ocular surface was rinsed with sterile PBS to remove residual tear fluid and debris. In one experimental group, a standardized Kimwipe™ blotting technique was used to gently wipe the corneal surface, first over the central cornea, followed by a rotational motion to include the periphery. Minimal pressure was applied to prevent mechanical injury while effectively reducing tear fluid, facilitating bacterial adhesion.

### Bacterial inoculum preparation and corneal inoculation

*Pseudomonas aeruginosa, Staphylococcus aureus* and *Staphylococcus epidermidis* were cultured on tryptic soy agar (TSA; Cat. No. 22091; Sigma-Aldrich, St. Louis, MO, USA) plates at 37 °C incubation overnight. Individual colonies were harvested, suspended in PBS, and centrifuged to remove non-viable cells and metabolic byproducts. The bacterial pellet was resuspended in PBS and adjusted to a final concentration of ~10¹¹ CFU/mL to ensure a high bacterial load for adhesion studies.

For corneal inoculation, a 5-μL aliquot of the bacterial suspension was applied to the blotted corneal surface every hour for 4 hours while maintaining sustained anesthesia. Mice were positioned at a slight incline to minimize runoff and ensure uniform bacterial exposure. After the inoculation period, mice were euthanized with an intraperitoneal injection of ketamine (80–100 mg/kg) and xylazine (5–10 mg/kg), followed by cervical dislocation. Eyes were enucleated under sterile conditions, rinsed with PBS to remove non-adherent bacteria, and fixed in 2% paraformaldehyde for one hour. The tissue was then homogenized in 1 mL of PBS, followed by serial six-fold dilutions. Diluted samples were plated on TSA at 37 °C incubation overnight to quantify bacterial load on the corneal surface and data were expressed in Log CFU.

### Immunohistochemistry

To evaluate corneal nerve density following euthanasia by cervical dislocation, eyes were carefully enucleated on day 15 post treatment and rinsed once with PBS at room temperature under gentle rotation. Corneas were then dissected and incubated in a blocking buffer containing 3% bovine serum albumin (BSA; Cat. No. A7906; Sigma-Aldrich, St. Louis, MO, USA) and Triton X-100 (Cat. No. X100; Sigma-Aldrich, St. Louis, MO, USA) diluted in PBS for one hour at room temperature with continuous rotation. This was followed by incubation in 20% Ethylenediaminetetraacetic acid (EDTA; Cat. No. E6511 Sigma-Aldrich, St. Louis, MO, USA) for another hour under the same conditions to facilitate tissue permeabilization. Subsequently, corneas were incubated overnight at 4°C in rotation with a primary antibody against Anti-β-Tubulin III antibody produced in rabbit (Cat. No. T2200; Sigma-Aldrich, St. Louis, MO, USA). The following day, tissues were incubated with a secondary antibody conjugated to Goat anti-Rabbit IgG secondary antibody, Alexa Fluor™ 488 (Cat. No. A-11008; Thermo Fisher Scientific, Waltham, MA, USA) and counterstained with DAPI (4’,6-Diamidino-2-Phenylindole, Dihydrochloride) (Cat. No. D1306; Thermo Fisher Scientific, Waltham, MA, USA) for nuclear visualization, all under foil-covered conditions at 4°C for 2–3 hours. After incubation, corneas were washed three times with PBS, each for 10 minutes with rotation, and then flat-mounted on glass slides using ProLong™ Gold Antifade Mountant (Cat. No. P36930; Thermo Fisher Scientific, Waltham, MA, USA) medium before cover slipping.

### Imaging

Confocal imaging was conducted using an upright Zeiss two-photon confocal microscope equipped with a 20 × /1.00 NA water-dipping objective. Flat-mounted corneas were imaged using a 488 nm laser (Alexa Fluor) to visualize corneal nerves and a 405 nm laser to label cell nuclei, with Z-stack images acquired at step sizes of 0.80 μm or 1.0 μm. Corneal nerve density image analysis was performed using Imaris (Bitplane). Imaging protocols followed previously established methods [26]. Where applicable, maximum intensity projections were used to compress 3D image data into 2D representations by projecting the highest intensity values from each pixel along the z-axis. Corneal nerve density under different experimental conditions was graded on a 0–10 scale by blinded examiners.

### ELISA assay

On day 15 post-bupivacaine treatment, mice were euthanized, and eyes were enucleated, corneas were dissected to remove limbal tissue, flash-frozen in liquid nitrogen, and homogenized. Protein extraction was done in 2% DL-Dithiothreitol (DTT; Cat. No. D0632; Sigma-Aldrich, St. Louis, MO, USA) in PBS from the pool of two to three corneas per condition, and protein concentration was normalized using the bicinchoninic acid assay (BCA; Cat. No. 23225; Thermo Fisher Scientific, Waltham, MA, USA). SP and CGRP protein levels were quantified using ELISA kits: Mouse SP ELISA Kit (Cat. No. MBS702782; MyBioSource, USA) and Mouse CGRP ELISA Kit (Cat. No. MBS452065; MyBioSource, USA), following the manufacturer’s protocols using extracted protein from mouse corneas and tear fluid.

### Statistical analysis

Data analysis was conducted using Prism 9.0 for Mac (GraphPad Software) and Microsoft Excel 2010. Distribution of data was assessed using Shapiro-Wilk and Kolmogorov-Smirnov tests. Since most of the acquired data was normally distributed, they were expressed as the mean and standard deviation (SD). Student’s t-test was used for two group comparisons and a one-way ANOVA with Tukey’s multiple comparisons was used for three or more groups. P values less than 0.05 were considered significant. All experiments were repeated three times unless otherwise stated.

## Results

### Corneal nerve density is significantly reduced following bupivacaine treatment

**Fig 2:**
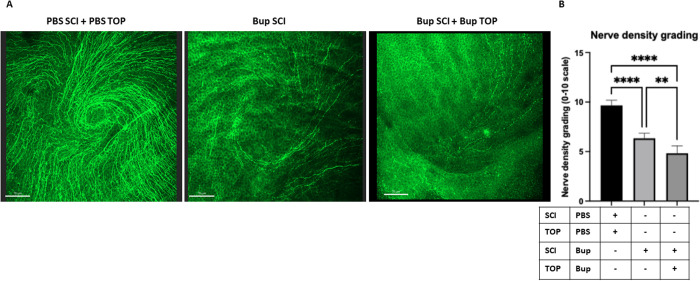
A. Representative confocal microscopy images of corneal nerves labeled with β-tubulin III (green) in WT mice treated with 0.5% bupivacaine (BUP) via subconjunctival injection (SCI) alone or in combination with topical (TOP) application, as well as PBS-treated controls receiving similar subconjunctival and topical applications. Corneal tissues were processed on day 15 post-injection. Scale bar = 70 µm. B. Quantification of corneal nerve density using a grading scale (0–10) assessed by a blinded researcher in WT mice across different treatment groups. A significant reduction in corneal nerve density was observed in mice treated with 0.5% bupivacaine (subconjunctival alone or in combination with topical application) compared to PBS-treated controls. Data are presented as mean ± SD. ** P < 0.01, **** P < 0.0001 (One-way ANOVA with Tukey’s multiple comparisons test).

To assess the effectiveness of targeted corneal sensory nerve suppression, we evaluated corneal nerve density on day 15 following alternate-day subconjunctival bupivacaine injections, either alone or in combination with topical application (Fig 1). Confocal microscopy of β-tubulin III-labeled corneal nerves revealed a substantial reduction in nerve density in both bupivacaine-treated groups compared to PBS-treated controls (Fig 2A). Blinded grading analysis confirmed a significant decrease in corneal nerve density in mice receiving subconjunctival bupivacaine alone (**P** < 0.01), with an even greater reduction observed in those receiving both subconjunctival and topical bupivacaine (**P** < 0.0001) (Fig 2B). Notably, the combined treatment resulted in the most pronounced corneal denervation, indicating deeper, more localized nerve suppression than subconjunctival injection alone, thereby demonstrating the efficacy of this approach in achieving sustained corneal sensory nerve suppression.

### Bupivacaine-Induced corneal denervation leads to reduced tear production

**Fig 3:**
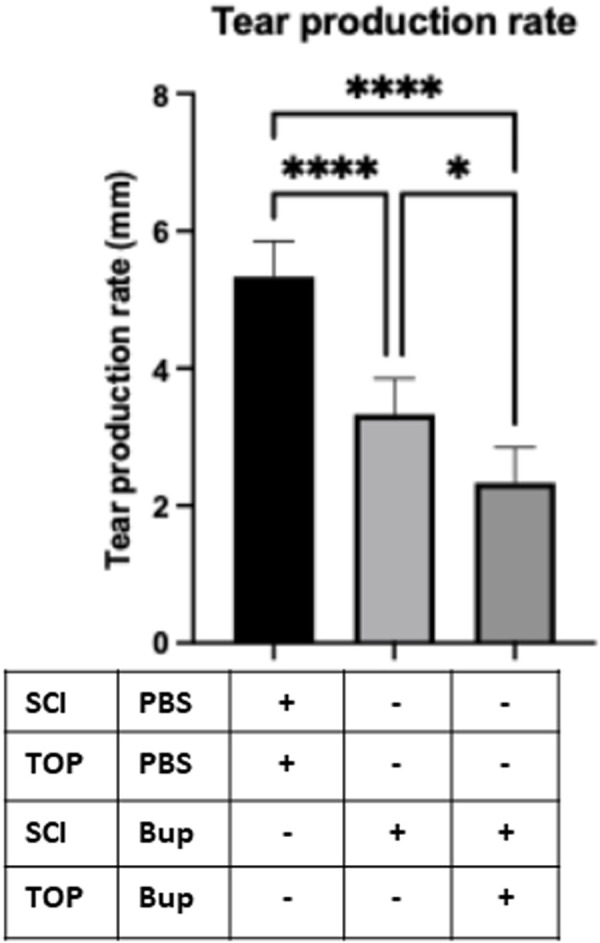
Tear production was quantified using the SMTube assay, revealing a significant reduction in tear secretion in mice treated with 0.5% bupivacaine (BUP), either via subconjunctival injection (SCI) alone or in combination with topical (TOP) application, compared to PBS-treated controls. The SMTube assay was performed four hours after the final BUP treatment on day 15 to assess aqueous tear volume. Data are presented as mean ± SD. * P < 0.05, **** P < 0.0001 (One-way ANOVA with Tukey’s multiple comparisons test).

Given the significant reduction in corneal nerve density following bupivacaine treatment, we next examined its functional impact on tear production. SMTube assay measurements revealed a notable decrease in tear production in both bupivacaine-treated groups compared to PBS-treated controls (Fig 3). Mice receiving subconjunctival bupivacaine alone exhibited a significant reduction in tear secretion (**P** < 0.05), while those treated with the combined subconjunctival and topical bupivacaine application demonstrated the most pronounced decline (**P** < 0.0001). These findings suggest that corneal sensory nerve suppression directly impairs tear production, with the combined treatment exerting a stronger inhibitory effect. Despite this marked reduction in tear secretion, corneal surface integrity remained unaffected, as no significant fluorescein staining changes were observed on day 15. These findings suggest that corneal sensory nerve suppression directly impairs tear production, with the combined treatment exerting a stronger inhibitory effect. The absence of corneal epithelial damage at this time point indicates that tear reduction alone may not immediately compromise corneal surface integrity.

### Corneal sensory nerve suppression reduces neuropeptide levels in the cornea and tear fluid

**Fig 4:**
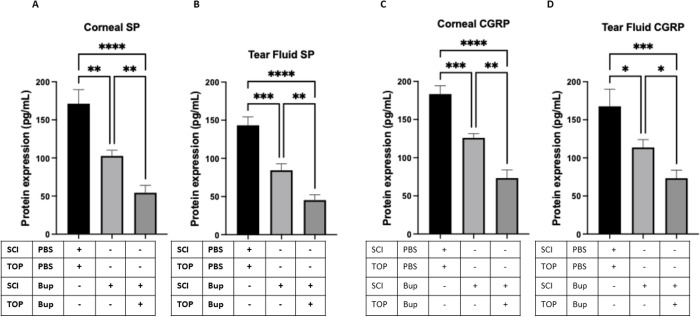
ELISA analysis revealed a significant decrease in SP (A & B) and CGRP (C & D) protein levels in the corneas and tear fluid of mice treated with 0.5% bupivacaine (BUP) every alternate day for 15 days (administered via subconjunctival injection (SCI) alone or in combination with topical (TOP) application) compared to PBS-treated controls. Protein levels were assessed on day 15 four hours post-injection and data are presented as mean ± SD. * P < 0.05, ** P < 0.01, *** P < 0.001, and **** P < 0.0001 [One-way ANOVA with Tukey’s multiple comparison test].

To further investigate the molecular consequences of corneal sensory nerve suppression, we analyzed the levels of neuropeptides implicated in tear production and immune modulation. ELISA quantification revealed a significant reduction in both SP and CGRP protein levels in the corneas and tear fluid of bupivacaine-treated mice compared to PBS-treated controls (Fig 4). Notably, the combined subconjunctival and topical bupivacaine treatment resulted in the most pronounced reduction, with **SP levels decreasing by ~3.16-fold** in both the cornea and tear fluid. Similarly, CGRP levels were significantly reduced, with a **~ 2.51-fold decrease in the cornea** and a **~ 2.29-fold decrease in the tear fluid** following the combined treatment. The subconjunctival-only group also exhibited a significant but less pronounced decrease. These findings indicate that corneal sensory nerve suppression diminishes neuropeptide availability, which may contribute to the observed impairment in tear secretion.

### Corneal sensory denervation enhances bacterial adhesion

Using this model, we investigated how corneal sensory nerve suppression influences bacterial adhesion dynamics for *P. aeruginosa*, *S. aureus*, and *S. epidermidis*—three clinically relevant pathogens with distinct adhesion mechanisms. To ensure uniform bacterial deposition, we employed the Kimwipe blotting method for standardized inoculation, followed by quantification of adhered bacteria.

**Fig 5:**
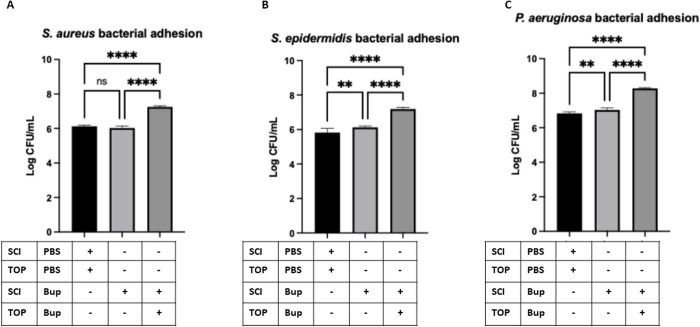
Quantification of bacterial adhesion showed a significant increase in adhered *S. aureus* (A), *S. epidermidis* (B), and *P. aeruginosa* (C) on blotted corneas of mice treated with 0.5% bupivacaine (BUP), either subconjunctival injection (SCI) alone or in combination with topical (TOP) application, compared to PBS-treated controls. The level of bacterial adhesion was assessed on Day 15 post-injection and data are presented as mean ± SD of Log CFU bacterial adhesion. ** P < 0.01, **** P < 0.0001, and ns = not significant (One-way ANOVA with Tukey’s multiple comparisons test).

Our results revealed that *S. aureus* adhesion was significantly increased in corneas treated with combined subconjunctival and topical bupivacaine compared to PBS-treated controls (Fig 5A). In contrast, *S. epidermidis* and *P. aeruginosa* exhibited significantly greater adhesion in both subconjunctival-alone and combined treatment groups (Fig 5B and 5C). Notably, *P. aeruginosa* displayed the highest overall adhesion levels compared to the Gram-positive bacteria. These findings suggest that the combined effect of subconjunctival bupivacaine injection with topical application, achieving both localized and sustained corneal sensory denervation, alters the ocular surface microenvironment. This alteration differentially enhances bacterial adhesion depending on the pathogen’s adhesion mechanisms.

### Reduction of bacterial adhesion with neuropeptide supplementation following sensory denervation

To determine whether exogenous neuropeptide supplementation could restore the altered neurogenic immune phenotype following sensory denervation, we administered CGRP to bupivacaine-treated mice, as CGRP-positive nerve fibers and terminals are more abundant than SP-positive ones [27] and assessed its effects on tear production and bacterial adhesion. Mice received subconjunctival and topical applications of 0.5% bupivacaine every alternate day for 14 days to achieve sustained corneal sensory denervation as discussed earlier (Fig 1). Then, in one experimental group, CGRP was administered via subconjunctival injection 24 hours before the final bupivacaine treatment (day 14) and again one hour prior to the final bupivacaine injection on day 15. Bacterial adhesions were assessed on day 15 to evaluate both the immediate and sustained effects of neuropeptide supplementation.

**Fig 6:**
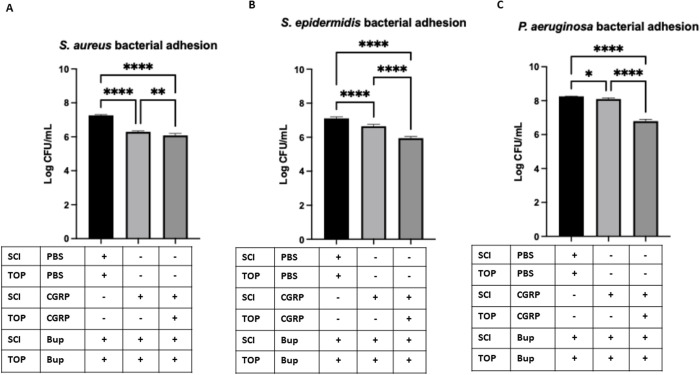
Quantification of bacterial adhesion revealed a significant decrease in the adhesion of *S. aureus*, *S. epidermidis*, and *P. aeruginosa* on blotted corneas of mice treated with 0.5% bupivacaine (BUP) combined with topical (TOP) application, following subconjunctival injection (SCI) of CGRP, 24 hours before the final BUP treatment (day 14) and again one hour prior to the final bupivacaine injection on day 15. The impact of neuropeptides on bacterial adhesion was assessed on day 15 and data are presented as mean ± SD of Log CFU bacterial adhesion. * P < 0.05, ** P < 0.01, **** P < 0.0001 (One-way ANOVA with Tukey’s multiple comparisons test).

Bacterial adhesion was significantly reduced in corneas of mice pre-treated with CGRP before the final bupivacaine administration. This effect was observed across all three tested bacterial strains—*S. aureus, S. epidermidis, and P. aeruginosa*—when compared to the PBS control group, which received PBS 24 hours before the final bupivacaine injection (Fig 6). These findings suggest that CGRP plays a critical role in maintaining corneal immune defense mechanisms, potentially by modulating the ocular surface microenvironment and limiting bacterial adhesion, while the role of SP in this context remains to be explored. The ability of these neuropeptides to counteract the adhesion-promoting effects of sensory denervation highlights their therapeutic potential in preventing infection-related complications following corneal nerve impairment.

### Sustained elevation of SP and CGRP in the cornea and tear fluid following neuropeptide supplementation

**Fig 7:**
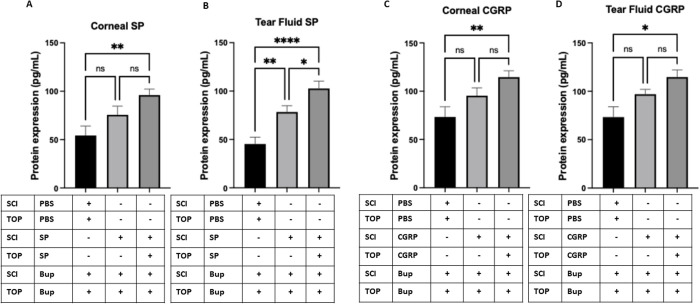
ELISA results showing SP (A & B) and CGRP (C & D) protein levels in mice treated with SP or CGRP via subconjunctival injection (SCI) and topical (TOP) application, 24 hours before the final bupivacaine (BUP) treatment (day 14) and again one hour prior to the final bupivacaine injection on day 15, compared to PBS-treated controls. Both SP and CGRP were significantly increased in corneas and tear fluid on day 15 post treatment. Data are presented as mean ± SD. * P < 0.05, ** P < 0.01, **** P < 0.0001, and ns = not significant (One-way ANOVA with Tukey’s multiple comparisons test).

To investigate the short-term impact of neuropeptide injection and the potential endogenous contribution to bacterial adhesion prevention, we quantified SP and CGRP levels in the cornea and tear fluid using ELISA. Mice receiving subconjunctival SP or CGRP injections on day 14 and 15 exhibited significantly elevated levels of the respective neuropeptides in both the cornea and tear fluid on day 15 compared to PBS-treated controls. Specifically, SP-treated mice showed a ~ **1.77-fold increase** in SP levels in the cornea and a ~ **2.26-fold increase** in the tear fluid, while CGRP-treated mice exhibited a ~ **1.56-fold increase** in CGRP levels in both compartments (Fig 7). This increase suggests that the injected neuropeptides remained bioavailable over an extended period and that their presence in the ocular surface milieu could have contributed to the observed restoration of corneal defense mechanisms.

## Discussion

In this study, we sought to better understand the impact of corneal sensory nerve suppression on ocular surface function and immune defense. Specifically, we aimed to explore how targeted sensory denervation, achieved through bupivacaine treatment, affects tear production, bacterial adhesion, and neuropeptide levels in the cornea and tear fluid. Our findings demonstrate that combined subconjunctival and topical bupivacaine resulted in deeper, more localized corneal nerve denervation compared to subconjunctival injection alone, which significantly reduced tear production without causing corneal epithelial damage. Additionally, this sensory denervation led to a decrease in SP and CGRP levels in both the cornea and tear fluid, which was most pronounced in the combined treatment group. Importantly, this suppression of neuropeptides coincided with an increase in bacterial adhesion, especially for *S. epidermidis* and *P. aeruginosa*, highlighting the role of corneal sensory nerves in modulating the ocular surface microenvironment. Exogenous supplementation of SP or CGRP restored neuropeptide levels and reduced bacterial adhesion, suggesting that these neuropeptides are critical for maintaining ocular immunity. These results underscore the importance of sensory nerves and neuropeptides in tear production and infection defense, providing insight into potential therapeutic approaches for addressing sensory nerve impairment in ocular diseases.

The pronounced reduction in corneal nerve density observed with the combined subconjunctival and topical bupivacaine treatment likely arises from the synergistic effects of these two delivery methods, resulting in more localized and deeper nerve suppression. Bupivacaine, a potent local anesthetic, blocks sodium channels, inhibiting nerve signal transmission [28–30]. When used in both subconjunctival injection and topical application, the anesthetic can diffuse more effectively across the corneal surface and into deeper nerve layers, providing a more comprehensive and localized denervation than either method alone. This enhanced method of anesthetic delivery aligns with studies showing that combined delivery methods improve drug penetration in ocular tissues [31]. The deeper and more profound nerve suppression observed in our study may thus reflect the additive pharmacokinetic advantages of dual administration. While we focused on corneal nerve density as a primary indicator of structural and functional integrity, future studies will incorporate additional clinically relevant parameters such as nerve length, tortuosity, and beading to enable a more comprehensive analysis.

The marked reduction in SP and CGRP directly correlates with the loss of corneal sensory nerves, which are essential for modulating inflammation, tear production, and immune defense on the ocular surface [32–34]. This decline aligns with previous reports that SP and CGRP stimulate lacrimal gland secretion and maintain tear film stability [35,36], providing a mechanistic link between sensory denervation and reduced tear secretion. Additionally, neuropeptide depletion has been associated with impaired antimicrobial defense, including reduced expression of antimicrobial peptides, which may explain the increased bacterial adhesion observed in our study [37]. While our findings highlight localized neuropeptide loss at the ocular surface, further investigation is needed to determine whether denervation disrupts central synthesis within the trigeminal ganglion or primarily affects peripheral release. This distinction is crucial for therapeutic strategies: if ganglionic synthesis is impaired, neurotrophic factor-based interventions may be necessary [38,39], whereas deficits in axonal transport or local release may be addressed through targeted delivery of neuropeptides or enhancers of peripheral nerve function. Clarifying these mechanisms could refine treatments for neurotrophic keratopathy and diabetic neuropathy, where both central and peripheral nerve dysfunction contribute to disease pathology. Our findings suggest that the combined treatment, by inducing more significant corneal denervation, leads to a more substantial reduction in SP and CGRP levels, further supporting the critical role of sensory nerves and neuropeptides in maintaining ocular surface homeostasis. The reduction in neuropeptide availability may impair the corneal epithelium’s ability to respond to microbial challenges, as SP and CGRP are known to enhance epithelial cell migration and upregulate defensins [40,41]. While our study focused on SP and CGRP as key neuropeptides associated with corneal sensory nerve function, future investigations utilizing multiplex assays such as Luminex may offer broader insights into the neuropeptide landscape.

Since the combined subconjunctival and topical bupivacaine treatment induces more profound corneal sensory denervation, leading to a substantial reduction in SP and CGRP levels, likely compromises the corneal epithelium’s ability to mount an effective defense against microbial challenges. SP and CGRP are known to enhance epithelial cell migration, upregulate defensins, and promote mucin secretion [40,41], all of which are essential for limiting bacterial adhesion. This mechanistic link is strongly supported by our observation that sensory denervation significantly increased adhesion of *S. aureus*, *S. epidermidis*, and *P. aeruginosa*—pathogens that exploit distinct surface receptors and adhesion strategies [14–17]. Notably, the heightened adhesion of *P. aeruginosa*, a Gram-negative pathogen with flagellar and biofilm-mediated virulence [14,15], suggests that neuropeptide depletion may disrupt mucin barrier integrity or impair epithelial signaling pathways that regulate surface glycoproteins targeted by bacterial adhesins.

The differential adhesion patterns observed—where *S. aureus* adhesion was most pronounced in the combined treatment group, while *S. epidermidis* and *P. aeruginosa* increased in both treatment groups—may reflect pathogen-specific interactions with neuropeptide-modulated defenses. For instance, *S. aureus* relies heavily on host surface proteins like fibronectin for adhesion, which are regulated by neuropeptide-driven epithelial repair mechanisms [42,43]. The restoration of bacterial adhesion resistance through exogenous CGRP supplementation directly implicates these neuropeptides in maintaining a hostile microenvironment for pathogens, likely through dual mechanisms: (1) enhancing epithelial barrier function and antimicrobial peptide production (e.g., β-defensins) and (2) modulating immune cell recruitment (e.g., neutrophils and macrophages) to neutralize adherent bacteria [18,20,21,39]. The efficacy of a single neuropeptide dose in mitigating adhesion, despite ongoing denervation, highlights their rapid therapeutic potential in counteracting infection risks associated with corneal nerve damage, such as in neurotrophic keratopathy or diabetic neuropathy. These findings reinforce the paradigm that sensory nerves and their neuropeptide mediators are indispensable gatekeepers of ocular surface immunity. While our study demonstrates that CGRP supplementation reduces bacterial adhesion, the exact mechanisms, along with the role of SP—whether through direct bactericidal activity, inhibition of bacterial adherence, or modulation of epithelial defense mechanisms—remain to be fully elucidated.

Our study demonstrated that combined subconjunctival and topical bupivacaine treatment reduced tear production, likely due to sensory denervation impairing neuropeptide-mediated lacrimal gland stimulation. While exogenous SP or CGRP supplementation restored tear secretion, we did not assess whether the sensory denervation extended beyond the cornea to affect lacrimal gland innervation. The lacrimal gland receives sensory and parasympathetic innervation from the trigeminal and facial nerves, respectively, with SP and CGRP playing key roles in modulating tear secretion [36,44]. Bupivacaine’s localized application may spare lacrimal gland nerves, but sustained corneal denervation could indirectly disrupt central trigeminal signaling to the lacrimal gland via axonal reflexes or neuropeptide depletion [45,46]. Additionally, retrograde tracing from the lacrimal gland to the trigeminal ganglion could clarify if the same sensory neurons innervate both the cornea and lacrimal gland [45,47,48]. Such work would elucidate whether tear suppression in our model stems from localized corneal denervation or broader trigeminal pathway dysfunction.

Overall, this study establishes a precise corneal denervation model using combined subconjunctival and topical bupivacaine, demonstrating that sensory nerve suppression reduces tear production and neuropeptides (SP/CGRP), leading to increased bacterial adhesion (*S. aureus, P. aeruginosa*). Exogenous SP/CGRP restored neuropeptide levels and reversed adhesion, underscoring their essential role in ocular immunity. Future studies should explore neuropeptide-based therapies for neurotrophic keratopathy, mechanisms of bacterial adhesion inhibition, and long-term nerve regeneration, advancing treatment strategies for neurogenic ocular diseases.

## Supporting information

S1 Table Supplementary data supporting the findings presented in the manuscript. (XLSX)
